# Diagnostic and Prognostic Value of MicroRNAs in Metastasis and Recurrence of Head and Neck Squamous Cell Carcinoma: A Systematic Review and Meta-Analysis

**DOI:** 10.3389/fonc.2021.711171

**Published:** 2021-09-27

**Authors:** Ke Qiu, Yao Song, Yufang Rao, Qiurui Liu, Danni Cheng, Wendu Pang, Jianjun Ren, Yu Zhao

**Affiliations:** ^1^ Department of Oto-Rhino-Laryngology, West China Hospital, Sichuan University, Chengdu, China; ^2^ West China Biomedical Big Data Center, West China Hospital/West China School of Medicine, Sichuan University, Chengdu, China; ^3^ Medical Big Data Center, Sichuan University, Chengdu, China

**Keywords:** microRNA, head and neck squamous cell carcinoma (HNSCC), recurrence-free survival (RFS), metastasis, diagnostic accuracy, meta-analysis

## Abstract

MicroRNAs have been proven to make remarkable differences in the clinical behaviors of head and neck squamous cell carcinoma (HNSCC). This study aims to systematically analyze whether differential expression levels of microRNAs are related to recurrence or metastasis in patients with HNSCC. A comprehensive search of the PubMed, EMBASE, and CENTRAL was conducted up to July 24^th^, 2021. Data were collected and combined from studies reporting recurrence-free survival (RFS) of HNSCC patients with high microRNA expression compared to those with low expression. Besides, studies providing necessary data for evaluating the diagnostic value of microRNAs for detecting recurrence and metastasis based on their expression levels were also included and combined. The pooled hazard ratio (HR) value for the outcomes of RFS in 1,093 HNSCC samples from 10 studies was 2.51 (95%CI: 2.13–2.96). A sensitivity of 0.79 (95% CI: 0.72–0.85) and specificity of 0.77 (95%CI: 0.68–0.83) were observed in three studies, of which 93 patients with recurrence and 82 nonrecurrence controls were included, and the area under the curve (AUC) was 0.85 (95% CI: 0.81–0.88). Additionally, high diagnostic accuracy of microRNAs in detecting lymph node metastasis (LNM) was also reported. In conclusion, two panels of microRNAs showed the potential to predict recurrence or diagnose recurrence in HNSCC patients, respectively, which could facilitate prognosis prediction and diagnosis of clinical behaviors in HNSCC patients.

**Systematic Review Registration:**

PROSPERO (https://www.crd.york.ac.uk/prospero), identifier CRD42020161117.

## Introduction

Head and neck squamous cell carcinomas (HNSCCs), visualized as the most common form of human solid tumors in the head and neck region, account for a large proportion of cancer mortality worldwide (1, 2). A multiple process that accumulates genetic mutations sequentially is believed to play a critical role in the formation of HNSCCs. And these highly heterogeneous tumors are derived from stratified epithelial cells of various anatomical subsites, mainly including the oral cavity, tongue, nasal cavity, larynx, and pharynx (3, 4). However, recent studies had illustrated similarities in genomic, genetic, and epigenetic alterations between HNSCCs from different subsites, which suggested the existence of certain common mechanisms underlying the initiation and progression of HNSCCs (3, 5, 6). It is estimated that approximately 1/3 of HNSCC patients develop recurrence or metastasis after receiving standard therapies, and the majority of them ended with poor prognosis (7, 8). Currently, tumor-node-metastasis (TNM) staging based on imaging modalities and biopsy represents the leading way to predict HNSCCs’ biological behaviors, especially for metastasis and recurrence; however, accuracy varied among HNSCCs with different origins (9, 10). Therefore, reliable and detectable biomarkers may contribute to the diagnosis and prediction of metastasis and recurrence of SCCs.

MicroRNAs, a special class of noncoding RNAs (19–23 nucleotides in length), are capable of binding to their target mRNAs and regulating gene expression at the post-transcriptional level (11). In the past decade, microRNAs had been proven to make remarkable differences in HNSCC carcinogenesis and cancer progression, making them potential biomarkers to predict biological behaviors of HNSCCs (12–18). This meta-analysis aimed at evaluating the diagnostic and prognostic values of microRNAs in the recurrence and metastasis of human SCCs.

## Methods and Materials

### Protocol and Eligibility Criteria

This study was conducted in accordance with the PRISMA (Preferred Reporting Items for Systematic Reviews and Meta-Analysis) guidelines (19) and was already registered on prospero (registration number: CRD42020161117).

The inclusion criteria were as follows: 1) studies written in English; 2) the diagnosis of HNSCC was confirmed by histopathology; 3) studies demonstrating the expression levels of microRNA by quantitative polymerase chain reaction (qPCR), *in situ* hybridization (ISH), fluorescent *in situ* hybridization (FISH), or RNA sequencing; 4) studies reporting HR with 95% confidence interval (CI) or Kaplan–Meier curves related to the correlation of microRNA expression with RFS; and 5) studies providing necessary data for evaluating the diagnostic value of microRNAs in predicting recurrence and metastasis.

The exclusion criteria were as follows: 1) studies related to nonhuman samples; 2) studies providing combined outcomes of more than one microRNA or insufficient data; 3) duplicate studies; 4) studies in the form of reviews, letters, editorials, meeting abstracts, or case reports; and 5) studies reporting SCC not originated from the head and neck region.

More specifically, for the meta-analysis evaluating the predictive values of microRNAs, we aimed to address the question of whether HNSCC patients with a higher expression level of microRNAs had different risk of cancer recurrence compared to HNSCC patients with a lower expression level of microRNAs. And for the meta-analysis evaluating the diagnostic values of microRNAs, we aimed to address the question of whether the expression level of microRNAs in human biospecimen could be used as indicators for distinguishing the HNSCC patients with recurrence/metastasis or not.

### Information Sources and Search Strategies

A comprehensive search of the PubMed, EMBASE, and CENTRAL was conducted from the beginning of each database to July 24^th^, 2021. Meanwhile, additional records from other sources, e.g., the references of included studies and original studies mentioned in reviews, were also screened.

A Boolean combination of Mesh terms and free text words were used as search strategies, mainly including “Carcinoma, Squamous Cell”[Mesh], “MicroRNA”, “MiRNAs”, “MicroRNAs”[Mesh], “Metastasis”, “Metastases”, “Recurrences”, “Relapse”, and “Recurrence”[Mesh]. Detailed search strategies were presented in **Supplementary Material**.

### Study Selection, Data Collection Process, and Data Items

The initial screening of titles and abstracts were conducted independently by two authors, and full texts would be reviewed if the titles and abstracts were ambiguous. Any discrepancy was solved by consensus, and a third author would participate if necessary. Finally, studies fulfilling all inclusion criteria were included in the systematic review.

Data extraction for studies investigating the RFS and diagnostic value was also independently performed and cross-checked by two authors. The agreement between authors KQ and YS was determined by Cohen’s kappa score. And the extracted items were as follows: year of publication, first author, country, microRNAs studied, type of microRNA dysregulation, sample sizes, duration of follow-up, tumor sites, metastasis sites, detection assay, sample types, and cutoffs for the expression of microRNAs.

### Risk of Bias Assessment, Summary Measures, and Statistical Analyses

The risk of bias within each included prognostic study was evaluated by the Newcastle-Ottawa Scale (NOS) (http://www.ohri.ca/programs/clinical_epidemiology/oxford.asp), and the risk of bias within each included diagnostic study was evaluated by the tool provided by Quality Assessment of Diagnostic Accuracy Studies—2 (QUADAS-2) (20), while the risk of bias across studies was evaluated by the Cochran Q test and Higgins index (*I^2^*). Heterogeneity was considered significant if *P* < 0.05 in Q test, and subgroup analyses were applied to find the potential sources of heterogeneity. Besides, overall effects were analyzed by a fixed-effect model if *I^2^* < 50%; otherwise, a random-effects model would be applied.

For studies investigating RFS, ln[HR] and standard error (SE) were synthesized (21). The publication bias was tested by Begg’s test. For studies investigating diagnostic values, we calculated the pooled sensitivity and specificity. The summary receiver operator characteristic (SROC) curve and the area under the SROC curve (AUC) were constructed and calculated to explore the diagnostic accuracy of microRNAs in metastasis/recurrence. Additionally, the publication bias was tested by Deek’s funnel plot asymmetry test.

The STATA 12.0 (Stata Corp, College Station, TX, USA) and Review Manager (Version5.4, The Cochrane Collaboration, 2020) software was used for all meta-analysis, and *P* < 0.05 was considered significant.

## Results

### Study Selection

**Figure 1:**
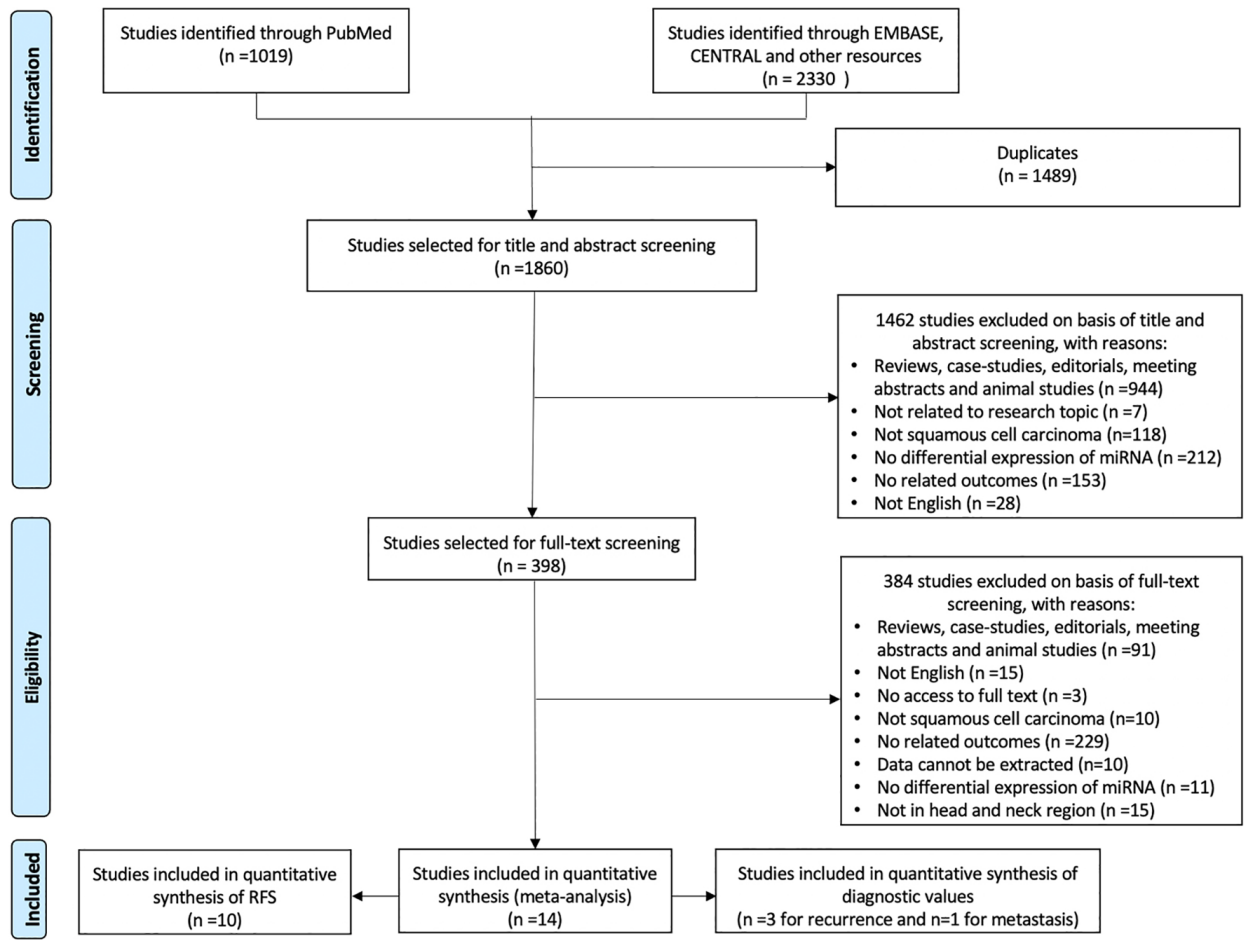
Flow diagram summarizing the selection process of the systematic review.

A total of 3,349 records were retrieved through the initial comprehensive search. A total of 1,860 articles remained after excluding duplicates and were screened according to titles and abstracts. Subsequently, 398 studies remained and underwent full-text screening. A total of 14 studies that fully met the inclusion criteria were included for further analysis, among which 10 studies (22–31) were included for meta-analysis of HR for RFS, 3 studies (32–34) were included for meta-analysis of diagnostic accuracy for recurrence, and another 1 study (14) was included for systematic review of diagnostic accuracy for metastasis. Detailed selection process and reasons of exclusion were presented in **Figure 1**.

### Study Characteristics

For the 10 included studies investigating RFS, a cumulative number of 1,093 SCC samples were analyzed. Tumor subsites were confined to head and neck regions, including the oral cavity, larynx, hypopharynx, and oropharynx. The duration of follow-up ranged from 28 months to more than 120 months, and all outcomes were evaluated by multivariate Cox regression models. Detailed characteristics of each study are presented in **Table 1**.

**Table 1:** Detailed characteristics and NOS scores of included studies investigating RFS.

Author and Year	Country		MicroRNA	Tumor sites and sample size	Assay	Type of samples	Dysregulation	Cutoff value	Follow-up time	Survival analysis
Ganci et al. (24)	Italy	miR-205-5p miR-429 miR-21-3p miR-331-3p miR-200a-3p miR-19a-3p miR-21-5p miR-151a-3p miR-17-3p miR-18b-5p miR-324-5p miR-96-5p		Oral cavity (n=73), larynx (n=29), hypopharynx (n=9) and oropharynx (n=10)	qPCR	Fresh frozen (tumor tissue)	Upregulation	According to special signal code	73M	Multivariate
Ganci et al. (25)	Italy	miR-429 miR-21-5p miR-96-5p miR-21-3p		Head and neck (n=106)	qPCR	FFPE (peritumor tissue)	Upregulation	According to special signal code	31M on average	Multivariate
Hudcova et al. (28)	Czech	miR-29c miR-200b miR-375		Head and neck (n=42)	qPCR	Fresh frozen (tumor tissue)	Downregulation	Not reported	30M	Multivariate
Bonnin et al. (23)	France	miR-422a		Oropharynx (n=75)	qPCR	Fresh frozen (tumor tissue)	Downregulation	Not reported	Unclear (more than 120M)	Multivariate
Ganci et al. (26)	Italy	miR-141-3p		Oral cavity (n=69)	qPCR	Fresh frozen (tumor tissue)	Upregulation	According to special signal code	50M	Multivariate
Harris et al. (27)	America	miR-375		Oral cavity (n=43)	qPCR	Fresh frozen (tumor tissue)	Downregulation	25-percentile	Unclear (more than 60M)	Multivariate
Harris et al. (27)	America	miR-375		Oropharynx (n=37)	qPCR	Fresh frozen (tumor tissue)	Downregulation	25-percentile	Unclear (more than 60M)	Multivariate
Harris et al. (27)	America	miR-375		Larynx (n=43)	qPCR	Fresh frozen (tumor tissue)	Downregulation	25-percentile	Unclear (more than 60M)	Multivariate
Ahmad et al. (22)	Czech	miR-15b-5p		Oral cavity (n=6), Hypopharynx (n=6), Larynx (n=8), Oropharynx (n=23)	qPCR	FFPE (tumor tissue)	Downregulation	Not reported	Unclear (more than 60M)	Multivariate
He et al. (29)	China	miR-130a		Oral cavity (n=92)	qPCR	Fresh frozen (plasma)	Upregulation	Median relative expression level	36M	Multivariate
Rajthala et al. (30)	Norway	miRNA-204		Oral cavity (n=158)	qPCR	FFPE (tumor tissue)	Downregulation	Not reported	8.6Y on average	Multivariate
Song et al. (31)	China	miR−200c		Oral cavity (n=204)	qPCR	Fresh frozen (tumor tissue)	Downregulation	Median relative expression level	36M	Multivariate

For the three included studies investigating diagnostic accuracy for recurrence, a total of 93 recurrence patients and 82 nonrecurrence controls were analyzed. Tumor subsites were confined to head and neck regions, including larynx and oral cavity. And the recurrence sites were not reported in most of the studies. Detailed characteristics of each study are presented in **Table 2**.

**Table 2:** Detailed characteristics of included studies investigating diagnostic values of microRNAs for recurrence and LNM.

Author and Year	Country	MicroRNA	Tumor sites and sample size	Assay	Type of samples	Number of samples		Metastasis/Recurrence sites
						M (+) OR R (+)	M (-) OR R (-)	
de Carvalho et al. (14)	Brazil	miR-200a miR-200c miR-203 miR-205	Head and neck (n=48)	qPCR	FFPE (lymph node)	25	23	Lymph node (metastasis)
Re et al. (32)	Italy	miR-34c-5p	Larynx (n=90)	qPCR	FFPE (tumor tissue)	49	41	Not reported (recurrence)
Re et al. (33)	Italy	miR-34c-5p	Larynx (n=43)	qPCR	FFPE (tumor tissue)	23	20	Not reported (recurrence)
Ries et al. (34)	Austria	miR-186-5p miR-3651 miR-494-5p	Oral cavity (n=42)	qPCR	Fresh frozen (whole blood)	21	21	Not reported (recurrence)

Similarly, for the included study investigating diagnostic accuracy for metastasis, a total of 25 metastasis patients and 23 nonmetastasis controls were analyzed. Tumor subsites were confined to head and neck regions. And the metastasis sites were reported to be the lymph node. Detailed characteristics of each study are presented in **Table 2**.

### Synthesis of RFS and Subgroup Analysis

**Figure 2:**
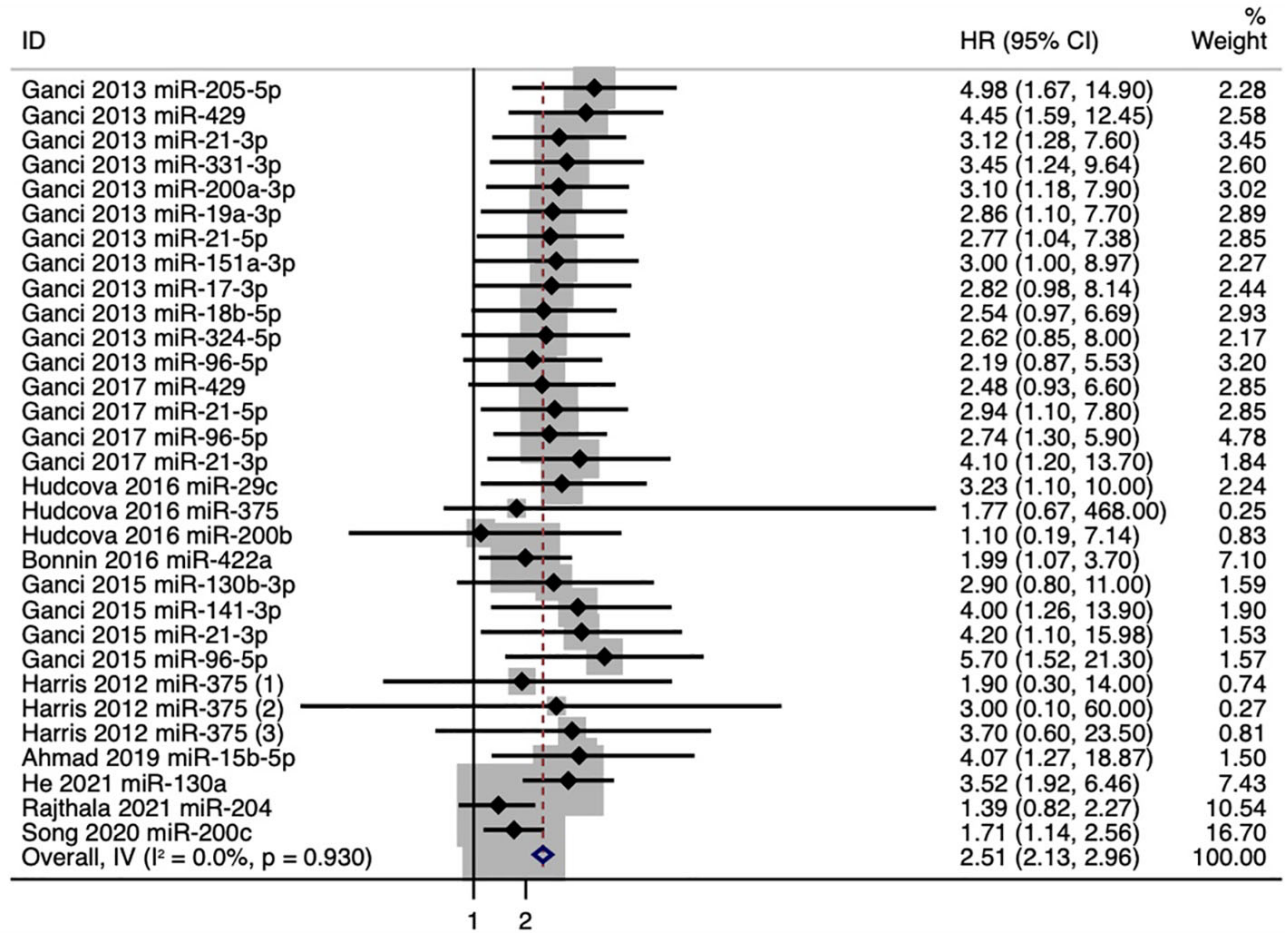
Forest plot for the association between microRNA expression and recurrence-free survival (RFS).

The pooled HR value for the outcomes of RFS in all HNSCC patients was 2.51 (95%CI: 2.13–2.96) (**Figure 2**). Poorer RFS correlated with upregulation of 14 microRNAs (miR-205-5p, miR-429, miR-21-3p, miR-331-3p, miR-200a-3p, miR-19a-3p, miR-21-5p, miR-151a-3p, miR-17-3p, miR-18b-5p, miR-324-5p, miR-96-5p, miR-141-3p, and miR-130a) and with downregulation of 7 microRNAs (miR-29c, miR-200b, miR-375, miR-422a, miR-15b-5p, miR-204, and miR-200c).

**Figure 3:**
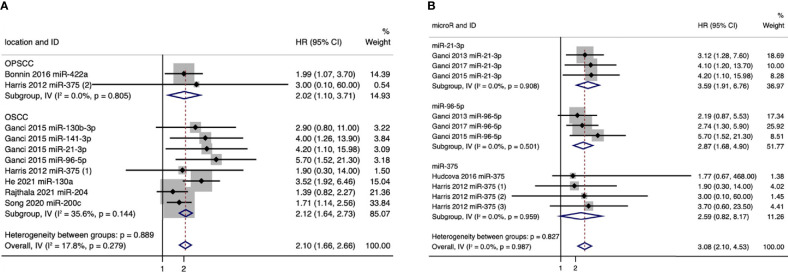
**(A)** Forest plot for subgroup analysis of the association between microRNA expression and recurrence-free survival (RFS) based on anatomical subsites. **(B)** Forest plot for subgroup analysis of the association between microRNA expression and recurrence-free survival (RFS) based on microRNAs (only individual microRNAs with more than two outcomes were presented).

We further conducted a subgroup analysis based on anatomical subsites, and the results showed that pooled HR values for the outcomes of RFS were 2.02 (95%CI: 1.10–3.71) in oropharyngeal squamous cell carcinoma (OPSCC) patients and 2.12 (95%CI: 1.64–2.73) in oral squamous cell carcinoma (OSCC) patients (**Figure 3**). And in OPSCC patients, poorer RFS correlated with downregulation of miR-422a and miR-375. While in OSCC patients, poorer RFS correlated with upregulation of five microRNAs (miR-21-3p, miR-130b-3p, miR-96-5p, miR-141-3p, and miR-130a) and with downregulation of three microRNAs (miR-375, miR-204, and miR-200c) (**Figure 3A**). Meanwhile, we also conducted a subgroup analysis for individual microRNAs with more than two outcomes. And the expression levels of miR-21-3p, miR-96-5p, and miR-375 showed strong association with RFS, especially miR-21-3p, with a pooled HR value of 3.59 (95%CI: 1.91-6.76) (**Figure 3B**).

Taken together, our results showed that a panel of 21 microRNAs (miR-205-5p, miR-429, miR-21-3p, miR-331-3p, miR-200a-3p, miR-19a-3p, miR-21-5p, miR-151a-3p, miR-17-3p, miR-18b-5p, miR-324-5p, miR-96-5p, miR-141-3p, miR-130a, miR-29c, miR-200b, miR-375, miR-422a, miR-15b-5p, miR-204, and miR-200c) might have the potential to predict the prognosis of patients with HNSCCs, of which 2 microRNAs were associated with the prognosis of OPSCC patients, and 8 microRNAs were associated with the prognosis of OSCC patients.

### Study Quality and Risk of Bias in Prognostic Studies

NOS scores of each prognostic study are listed in **Table S1**, of which eight studies (80%) were of “good” quality and deemed to have low risk of bias, while the other two studies (20%) were of “poor” quality and deemed to have high risk of bias mainly due to the lack of adjustment for important confounding variables. A Cohen’s kappa score of 0.76 revealed great agreement beyond chance between the two authors. Besides, no statistically significant heterogeneity (*P* > 0.1) and publication bias (Begg’s test: *P* = 0.333, **Figure S1**) was observed in the pooled analysis of RFS.

### Pooled Diagnostic Accuracy of microRNAs for HNSCC Recurrence

**Figure 4:**
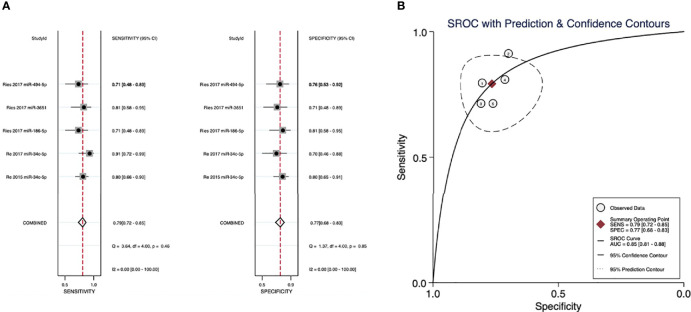
**(A)** Sensitivity and specificity of microRNAs in diagnosing recurrence. **(B)** The summary receiver operating characteristic (SROC) curves of the diagnostic performance of microRNAs for recurrence.

The evaluation for the sensitivity and specificity of a panel of four microRNAs in diagnosing the recurrence of SCCs is illustrated in **Figure 4A** (miR-34c-5p, miR-186-5p, miR-3651, and miR-494-5p). A sensitivity of 0.79 (95% CI: 0.72–0.85) and a specificity of 0.77 (95% CI: 0.68–0.83) were observed in patients with recurrence and nonrecurrence controls. The AUC was 0.85 (95% CI: 0.81–0.88), and the corresponding SROC curve is presented in **Figure 4B**.

### Study Quality and Risk of Bias in Diagnostic Studies

As is shown in **Figure S2**, nearly all included diagnostic studies showed low risk of bias in patient selection, reference standard, and flow and timing; however, all of these three studies showed high risk of bias in index test mainly due to their retrospective nature, in which the index test results cannot be interpreted without knowledge of the results of the reference standard. Besides, the diagnostic thresholds in these studies were not confined and also not prespecified, which might cause some concerns on applicability. Meanwhile, no significant heterogeneity (*P* > 0.1) and publication bias (*P* = 0.53, **Figure S3**) was observed.

### Overview of Diagnostic Accuracy of microRNAs for LNM in HNSCC

Only one study reporting the diagnostic accuracy of four independent microRNAs for LNM in HNSCC met our eligibility criteria. However, given the high risk of bias for pooled analysis of four tests derived from the same study, meta-analysis was not conducted. de Carvalho et al. reported the diagnostic accuracy of miRNA-200a (sensitivity 0.76; specificity:0.88; AUC: 0.92), miRNA-200c (sensitivity: 0.88; specificity: 1.00; AUC: 0.94), miRNA-203 (sensitivity: 1.00; specificity: 1.00; AUC: 1.00), and miRNA-205 (sensitivity: 1.00; specificity: 1.00; AUC: 1.00) in distinguishing 25 HNSCC patients with LNM from 23 HNSCC patients without LNM (14). All of these four microRNAs showed high diagnostic accuracy for detecting LNM in HNSCC patients; however, their performance was evaluated in the same cohort of which the sample size is not large enough. Thus, whether microRNAs have the potential for detecting LNM in HNSCC still needs to be validated by more studies with larger sample sizes.

## Discussion

In this review, we systematically analyzed 1,093 HNSCC samples from 10 studies (22–31) and identified a panel of 21 microRNAs related to poor RFS in HNSCC patients for the first time. Besides, we investigated the diagnostic accuracy of microRNAs for recurrence (by analyzing 93 recurrence patients and 82 nonrecurrence controls from 3 studies) (32–34) and LNM (by presenting an overview of 25 metastasis patients and 23 nonmetastasis controls from another study) (14). We observed relatively high diagnostic accuracy of microRNAs in diagnosing recurrence and LNM for HNSCCs, which have the potential to assist imaging modalities and histopathology biopsy in the diagnosis and prognosis for HNSCC patients.

In the current study, we specifically took recurrence and metastasis as the outcomes. Our results showed that a panel of 21 microRNAs might be suitable biomarkers for predicting the recurrence of HNSCCs (pooled HR:2.51, 95%CI: 2.13–2.96, *I^2 =^* 0), among which poor RFS correlated with upregulation of 14 microRNAs (miR-205-5p, miR-429, miR-21-3p, miR-331-3p, miR-200a-3p, miR-19a-3p, miR-21-5p, miR-151a-3p, miR-17-3p, miR-18b-5p, miR-324-5p, miR-96-5p, miR-141-3p, and miR-130a) or with downregulation of 7 microRNAs (miR-29c, miR-200b, miR-375, miR-422a, miR-15b-5p, miR-204, and miR-200c). Besides, subgroup analyses revealed similar trends in OSCC and OPSCC patients as well as identified miR-21-3p, miR-96-5p, and miR-375, which showed strong association with RFS. Our quality assessments showed that 80% of the included studies investigating RFS were of good quality, and the meta-analysis was of low risk of bias, which reinforced the reliability of our results. Meanwhile, the diagnostic accuracy of microRNAs in HNSCC recurrence was decent (sensitivity: 0.79, 95% CI: 0.72–0.85; specificity: 0.77, 95%CI: 0.68–0.83; AUC: 0.85, 95% CI: 0.81–0.88), together with a low rate of heterogeneity and low risk of publication bias. Additionally, the diagnostic accuracy of microRNAs in LNM of HNSCC reported by de Carvalho et al. was relatively high but needs further validation.

We further summarized these analyzed microRNAs and explored their well-known biological functions related to tumor malignant behaviors in **Table S2**. As expected, the downregulation of microRNAs, which had been previously reported to be related to tumor suppressing functions, including inhibiting tumor cell proliferation, migration, and invasion (18, 35–61), was associated with poorer RFS in our study. Similarly, the upregulation of microRNAs, which had been previously reported to function as enhancers of tumor malignant behaviors (62–83), were associated with poorer RFS. Besides, the modulation of epithelial to mesenchymal transition (EMT), an essential early step of tumor metastasis, was identified as the most commonly reported mechanism, through which those microRNAs regulate malignant behaviors of other types of tumor. Meanwhile, surprisingly, we found five members of the microRNA-200 family (miR-200a, miR-200b, miR-200c, miR-429, and miR-141), which are known for their regulatory function on EMT, and their combination might act as a strong prognosis predictor (**Figure S3**) (39, 84–88). Thus, we hypothesized that the modulation of EMT and subsequent cell migration and invasion might also act as the key pathophysiological mechanisms of the interplay between microRNAs and HNSCC, which still needs to be further confirmed by mechanistical studies.

There were some limitations in this study. First, given the fact that most of the included studies performed their analyses in HNSCC samples with mixed subsites, which cannot be strictly separated, therefore subgroup analysis could only be available in OSCC and OPSCC patients. And consequently, whether our conclusions can be generalized to SCC derived from other subsites remained to be verified by further studies with larger sample sizes and a wider spectrum of HNSCC subsites. Second, it is also a pity that most of these included studies have not performed subgroup analyses based on HPV status; thus, we were not able to properly investigate the influence of the HPV status. However, most of these included studies have adjusted the HPV status in their multivariate analysis, which might reduce its influence to some extent. Third, the outcomes of four independent microRNAs for LNM in HNSCC were retrieved from the same study. Thus, pooled analysis was not conducted due to the potential high risk of bias. And the reported high diagnostic accuracy still needs to be further validated. Additionally, our results should be explained cautiously since our panels have significance when these microRNAs are taken as a whole, not individually.

## Data Availability Statement

The datasets generated for this study are available on request to the corresponding authors.

## Author Contributions

JR and YZ contributed to the study conception and design. Material preparation was performed by QL and WP, data collection was performed by YR, and analysis was performed by KQ, YS, YR, and DC. The first draft of the manuscript was written by KQ and YS, and all authors commented on previous versions of the manuscript. All authors contributed to the article and approved the submitted version.

## Funding

This work was supported by West China Hospital, Sichuan University (YZ, grant # 2019HXFH003, grant # ZYJC21027); Chengdu Science and Technology Bureau (JR, grant # 20GJHZ0193); Sichuan University (YZ, grant # 20ZDYF3010, RJJ, grant # 2019HXBH079, #2020SCU12049); The Science and Technology Department of Sichuan Province (YZ, grant # 2020YFH0090, RJJ, grant#2020YFS0111); The Health Department of Sichuan Province (JR, grant # 20PJ030); China Postdoctoral Science Foundation (JR, grant # 2020M673250); The Foundation of National Clinical Research Center for Geriatrics (SH, grant # Z20201013); and National Natural Youth Science Foundation of China (JR, grant # 82002868).

## Conflict of Interest

The authors declare that the research was conducted in the absence of any commercial or financial relationships that could be construed as a potential conflict of interest.

## Publisher’s Note

All claims expressed in this article are solely those of the authors and do not necessarily represent those of their affiliated organizations, or those of the publisher, the editors and the reviewers. Any product that may be evaluated in this article, or claim that may be made by its manufacturer, is not guaranteed or endorsed by the publisher.
