# Supplementary material for: Diagnostic and Prognostic Value of MicroRNAs in Metastasis and Recurrence of Head and Neck Squamous Cell Carcinoma: A Systematic Review and Meta-Analysis
Source: Front Oncol. 2021 Sep 27;11:711171. doi: 10.3389/fonc.2021.711171 (PMC8503605; doi:10.3389/fonc.2021.711171)
Supplement: Supplementary file 1 [file DataSheet_1.docx]

*Supplementary materials 1:*

Detailed search strategies:

(((("Carcinoma, Squamous Cell"[Mesh]) OR (((((((((((((((Carcinomas, Squamous Cell) OR Squamous Cell Carcinomas) OR Squamous Cell Carcinoma) OR Carcinoma, Squamous) OR Carcinomas, Squamous) OR Squamous Carcinoma) OR Squamous Carcinomas) OR Carcinoma, Epidermoid) OR Carcinomas, Epidermoid) OR Epidermoid Carcinoma) OR Epidermoid Carcinomas) OR Carcinoma, Planocellular) OR Carcinomas, Planocellular) OR Planocellular Carcinoma) OR Planocellular Carcinomas))) AND (((((((((((((((((((MicroRNA) OR miRNAs) OR Micro RNA) OR RNA, Micro) OR miRNA) OR Primary MicroRNA) OR MicroRNA, Primary) OR Primary miRNA) OR miRNA, Primary) OR pri-miRNA) OR pri miRNA) OR RNA, Small Temporal) OR Temporal RNA, Small) OR Small Temporal RNA) OR pre-miRNA) OR pre miRNA)) OR "MicroRNAs"[Mesh])) AND ((((Metastasis OR metastases))) OR (((((((Recurrences) OR Recrudescence) OR Recrudescences) OR Relapse) OR Relapses)) OR "Recurrence"[Mesh])).

Table S1. Quality assessment of the included studies in the synthesis of RFS based on NOS scale

| Author | Year | Selection | | | | Comparability | Outcome | | | Quality rating |
| --- | --- | --- | --- | --- | --- | --- | --- | --- | --- | --- |
|  |  | Representativeness of exposed cohort | Representativeness of the unexposed cohort | Ascertainment of exposure | Demonstration, outcome of the study, was not present at the beginning of the study | Comparability of cohorts | Assessment of outcome | Follow-up long enough | Adequacy of follow-up cohorts |  |
| Ganci | 2013 | * | * | * | * | * | * | * | * | Good |
| Ganci | 2017 | * | * | * | * | * | * | * | * | Good |
| Hudcova | 2016 | * | * | * | - | - | * | * | * | Poor |
| Bonnin | 2016 | * | * | * | * | - | * | * | * | Good |
| Ganci | 2015 | * | * | * | * | * | * | * | * | Good |
| Harris | 2012 | * | * | * | * | ** | * | * | * | Good |
| Ahmad | 2019 | * | * | * | - | * | * | * | * | Good |
| He | 2021 | * | * | * | * | ** | * | * | * | Good |
| Rajthala | 2021 | * | * | * | - | - | * | * | * | Poor |
| Song | 2020 | * | * | * | - | ** | * | * | * | Good |

Table S2. Summary of analyzed microRNAs and their biological function related to tumor metastasis, recurrence or progression

| MicroRNA | Target gene | Tumor type | Biological function related to tumor metastasis, recurrence or progression | Reference |
| --- | --- | --- | --- | --- |
| miR-205-5p | VEGFA | Bladder cancer | Accelerating cell propagation, migration and invasiveness | (Cao et al., 2019) |
|  | ERBB | Breast cancer | Promoting tumor growth and metastatic spreading | (De Cola et al., 2018) |
|  | ZEB1 | Colon cancer | Inhibiting epithelial to mesenchymal transition (EMT), impairing cancer cell migration and restoring the epithelial phenotype | (Gulei et al., 2018) |
|  | TIMP‑2 | OSCC | Suppressing pro‑MMP‑2 activation and inhibiting OSCC cell invasiveness | (Nagai et al., 2018) |
|  | ZEB1 | Clear cell renal cell carcinoma | Modulating of EMT and tumor metastasis. | (Xiang et al., 2020) |
|  | PTEN | Nasopharyngeal carcinoma | Regulating EMT | (Zhang et al., 2019) |
| miR-429 | ZEB1 and SIP1 | Breast cancer | Inhibiting EMT | (Gregory et al., 2008) |
|  | CRKL | Hepatocellular carcinoma | Suppressing tumor migration and invasion via inhibiting Raf/MEK/ERK pathway and EMT | (Guo et al., 2018) |
|  | AKT1 | Melanoma | Inhibiting cancer cell proliferation and migration | (Huang et al., 2019) |
|  | PTEN, RASSF8 and TIMP2 | Non-small cell lung cancer (NSCLC) | Promoting cell proliferation, migration and invasion | (Lang et al., 2014) |
|  | BMI1 and E2F3 | Renal cell carcinoma | Suppressing cell proliferation, EMT, and metastasis | (Qiu et al., 2015) |
|  | KIAA0101 | Soft tissue sarcomas | Inhibiting metastasis | (Samantarrai and Mallick, 2017) |
|  | Onecut2 | Colorectal carcinoma | Inhibiting cell growth and invasion | (Sun et al., 2014) |
|  | ZEB1 | Thyroid cancer | Suppressing cell growth and inducing apoptosis | (Wu et al., 2019) |
|  | RAB23 | Hepatocellular carcinoma | Decreasing the migratory capacity and reversing the EMT to MET | (Xue and Tian, 2018) |
|  | CRKL and MMP-9 | Breast cancer | Inhibiting bone metastasis | (Zhang et al., 2020b) |
| miR-21-3p | L1CAM | Renal, endometrial and ovarian carcinoma | Promoting cell motility, invasion, chemoresistance and metastasis formation | (Doberstein et al., 2014) |
|  | - | Oral cancer | Promoting metastasis | (Tseng et al., 2017) |
| miR-331-3p | PHLPP | Hepatocellular carcinoma | Promoting cell proliferation and EMT-mediated metastasis | (Chang et al., 2014) |
|  | ST7L | pancreatic cancer | Promoting cell proliferation and EMT-mediated metastasis | (Chen et al., 2018a) |
|  | ErbB2 and VAV2 | NSCLC | Suppressing EMT, migratory capacity, and metastatic ability | (Li et al., 2019a) |
|  | NRP2 | Breast cancer | Inhibiting cell proliferation and increasing cell apoptosis | (Zhao et al., 2020) |
| miR-200a-3p | IRS2 | NSCLC | Suppressing cell proliferation, migration and invasion | (Tan et al., 2020) |
|  | ZEB1 | Lung cancer | Promoting invasion and metastasis | (Sarkar et al., 2020) |
| miR-19a-3p | PMEPA1 | Prostate cancer | Inducing cell proliferation, migration and invasion | (Feng et al., 2016) |
|  | SOX4 | Prostate cancer | Suppressing invasion and metastasis | (Feng et al., 2018) |
|  | PTEN | Hepatocellular carcinoma | Promoting tumor metastasis and chemoresistance | (Jiang et al., 2018b) |
|  | SMAD2 and SMAD4 | Prostate cancer | Suppressing invasion, migration and bone metastasis | (Wa et al., 2018) |
|  | Fra-1 | Breast cancer | Inhibiting cancer progression and metastasis | (Yang et al., 2014) |
| miR-21-5p | SMAD7 | Gastric cancer | Promoting peritoneal metastasis via mesothelial-to-mesenchymal transition | (Li et al., 2018b) |
|  | PDCD4 | Osteosarcoma | Promoting cell growth and metastasis | (Zhang and Xia, 2017) |
|  | SMAD7 | Lung adenocarcinoma | Promoting cell growth and metastasis | (Zhu et al., 2020) |
| miR-151a-3p | P53 | Nasopharyngeal carcinoma | Inducing cell proliferation, migration and invasion | (Liu et al., 2019a) |
|  | RAB22A | Osteosarcoma | Inhibiting invasive and migratory potentials of osteosarcoma cells | (Zheng et al., 2019) |
| miR-17-3p | Par4 | Colon cancer | Promoting cell proliferation and survival | (Lu et al., 2018) |
|  | TIMP3 | Prostate tumor | induce prostate tumor growth and invasion | (Yang et al., 2013) |
| miR-18b-5p | DOCK4 | Breast cancer | Promoting cell invasion, metastasis, and EMT | (Wang et al., 2019b) |
| miR-324-5p | ETS1 and SP1 | Hepatocellular Carcinoma | Inhibiting cancer migration and invasion | (Cao et al., 2015) |
|  | ELAVL1 | Colorectal Cancer | Inhibiting cell proliferation and invasion | (Gu et al., 2019) |
|  | SP1 | Cervical cancer | Inhibiting cell colony formation, proliferation, migration, invasion and EMT progression | (Jiang et al., 2018a) |
|  | TSPAN8 | Gastric cancer | Reducing cell viability and inducing apoptosis | (Lin et al., 2018) |
|  | PTPRD | Papillary thyroid carcinoma | Promoting cancer progression | (Yang et al., 2020b) |
|  | TGFB2 | Gallbladder carcinoma | Inhibiting cell metastatic behaviors | (Zhang et al., 2020c) |
| miR-96-5p | CCDC67 | Papillary thyroid carcinoma | Promoting cell proliferation, invasion and metastasis | (Liu et al., 2019b) |
|  | RASSF8 | NSCLC | Promoting EMT and metastasis | (Wei et al., 2019b) |
|  | SFRP4 | Cervical Cancer | Facilitating cell viability, migration, and invasion and suppressing cell apoptosis | (Zhang et al., 2020a) |
| miR-141-3p | GP73 | Hepatocellular carcinoma | Reversing EMT, subsequently inhibiting cancer progression and metastasis | (Hou et al., 2019) |
|  | - | NSCLC | Inhibiting cancer proliferation and metastasis | (Lu and Zhang, 2020) |
| miR-130a | PTEN | Osteosarcoma | Promoting metastasis and EMT | (Chen et al., 2016) |
|  | FOSL1 | Breast cancer | Suppressing cancer cell migration and invasion | (Chen et al., 2018b) |
|  | HIF1A | NSCLC | Inhibiting tumor metastasis | (Shi et al., 2020) |
|  | TBL1XR1 | Gastric carcinoma | Suppressing cell migration and invasion by inhibition of EMT | (Wang et al., 2018) |
|  | ZEB1 | Osteosarcoma | Inhibiting cell growth and metastasis | (Yi et al., 2017b) |
| miR-29c | TIAM1 | Nasopharyngeal carcinoma | Suppressing cancer invasion and metastasis | (Liu et al., 2013) |
|  | Integrin β1 and MMP2 | lung cancer | Suppressing cancer cell adhesion to extracellular matrix and metastasis | (Wang et al., 2013) |
|  | KIAA1199 | Gastric cancer | Inhibiting metastasis by deactivating EMT-related signaling pathways | (Wang et al., 2019a) |
|  | PTP4A and GNA13 | Colorectal carcinoma | Suppressing cell migration and invasion abilities | (Zhang et al., 2014) |
| miR-200b | MMP9 | Cervical carcinoma | Suppressing cell invasion and metastasis by inhibiting the EMT | (Cheng et al., 2016) |
|  | ARHGAP18 | Breast cancer | Suppressing metastasis | (Humphries et al., 2017) |
|  | ZEB2 | Glioma | Inhibiting tumor growth and metastasis | (Li et al., 2016) |
|  | LAMA4 | renal cell carcinoma | Suppressing metastasis | (Li et al., 2019b) |
|  | - | Prostate cancer | Inhibiting cancer EMT, growth and metastasis | (Williams et al., 2013) |
|  | FOXG1 | Cervical cancer | Promoting cell proliferation and metastasis | (Zeng et al., 2016) |
| miR-375 | PDGFA | OSCC | Inhibiting cell migration and invasion | (Cao et al., 2017) |
|  | SP1 | Colorectal cancer | Inhibiting cell invasion and metastasis | (Cui et al., 2016) |
|  | - | HNSCC | Suppressing extracellular matrix degradation and invadopodial activity | (Jimenez et al., 2015) |
|  | IGF1R | Esophageal squamous cell carcinoma (ESCC) | Inhibiting tumor growth and metastasis | (Kong et al., 2012) |
|  | SP1 | Cervical cancer | Inhibiting cell migration and invasion | (Wang et al., 2011) |
|  | RECK | Colorectal cancer | Accelerating cell invasion and migration | (Wei et al., 2019a) |
|  | PAX2 | Ovarian cancer | Inhibiting the growth, drug sensitivity and metastasis | (Yang et al., 2019) |
|  | SHOX2 | ESCC | Suppressing tumor invasion and metastasis | (Yi et al., 2017a) |
| miR-422a | TGFβ2 | Osteosarcoma | Inhibiting cell proliferation and invasion | (Liu et al., 2016) |
|  | RPN2 | Glioma | Suppressing cell proliferation and invasion, and inducing cell apoptosis | (Sun et al., 2020) |
|  | IGF1 and IGF1R | Glioma | Inhibiting cell proliferation and invasion | (Wang et al., 2017) |
|  | FOXG1, FOXQ1, and FOXE1 | Hepatocellular carcinoma | Inhibiting tumor cell proliferation and migration | (Zhang et al., 2015) |
| miR-15b-5p | MYCN | Neuroblastoma | Inhibiting tumor progression | (Chava et al., 2020) |
|  | PAQR3 | Gastric cancer | Promoting cancer metastasis | (Zhao et al., 2017) |
| miR-204 | FOXC1 | Endometrial cancer | Repressing cell migration, invasion and extracellular matrix-adhesion | (Chung et al., 2012) |
|  | PI3K | Breast cancer | Inhibiting cell proliferation and metastasis | (Fan et al., 2019) |
|  | SOX4 | Lung adenocarcinoma | Inhibiting tumor metastasis | (Hu et al., 2019) |
|  | NUAK1 | NSCLC | Inhibiting tumor metastasis | (Shi et al., 2014) |
|  | HER‑2 | Gastric cancer | Promoting cell apoptosis and inhibiting cell migration | (Yang et al., 2020a) |
|  | NUAK1 | Hepatocellular cancer | Inhibiting tumor metastasis | (Yu et al., 2019) |
|  | Notch2 | Gallbladder cancer | Inhibiting cell proliferation, invasion and apoptosis | (Zhang et al., 2021) |
| miR-200c | ZEB2 | Breast cancer | Attenuating cell invasion and EMT | (Chen et al., 2020) |
|  | ZEB1, ETS1 and FLT1 | Colorectal cancer | Mediating EMT and metastatic behavior | (Hur et al., 2013) |
|  | HMGB1 | Lung cancer | Inhibiting EMT, invasion, and migration | (Liu et al., 2017) |
|  | MAP4K4 | Cervical cancer | Suppressing cell metastasis and growth | (Mei et al., 2018) |
|  | ZEB1 | OSCC | Suppressing tumor metastasis by inhibiting EMT | (Xie et al., 2018) |
| miR-34c-5p | SATB2 | Colorectal cancer | Suppressing proliferation and metastasis by attenuating the EMT | (Gu et al., 2018) |
| miR-186-5p | PTEN | Lung adenocarcinoma | Promoting cell growth, migration and invasion | (Feng et al., 2019) |
|  | ZEB1 | Colorectal cancer | Inhibiting cell proliferation, metastasis and EMT | (Li et al., 2018a) |
|  | MORC2 | Cholangiocarcinoma | Inhibiting cell growth and metastasis | (Liao et al., 2019) |
| miR-3651 | TBX1 | Colorectal cancer | Promoting cell proliferation | (Li et al., 2020) |
| miR-494-5p | Not identified | Not identified | Not identified | Not identified |


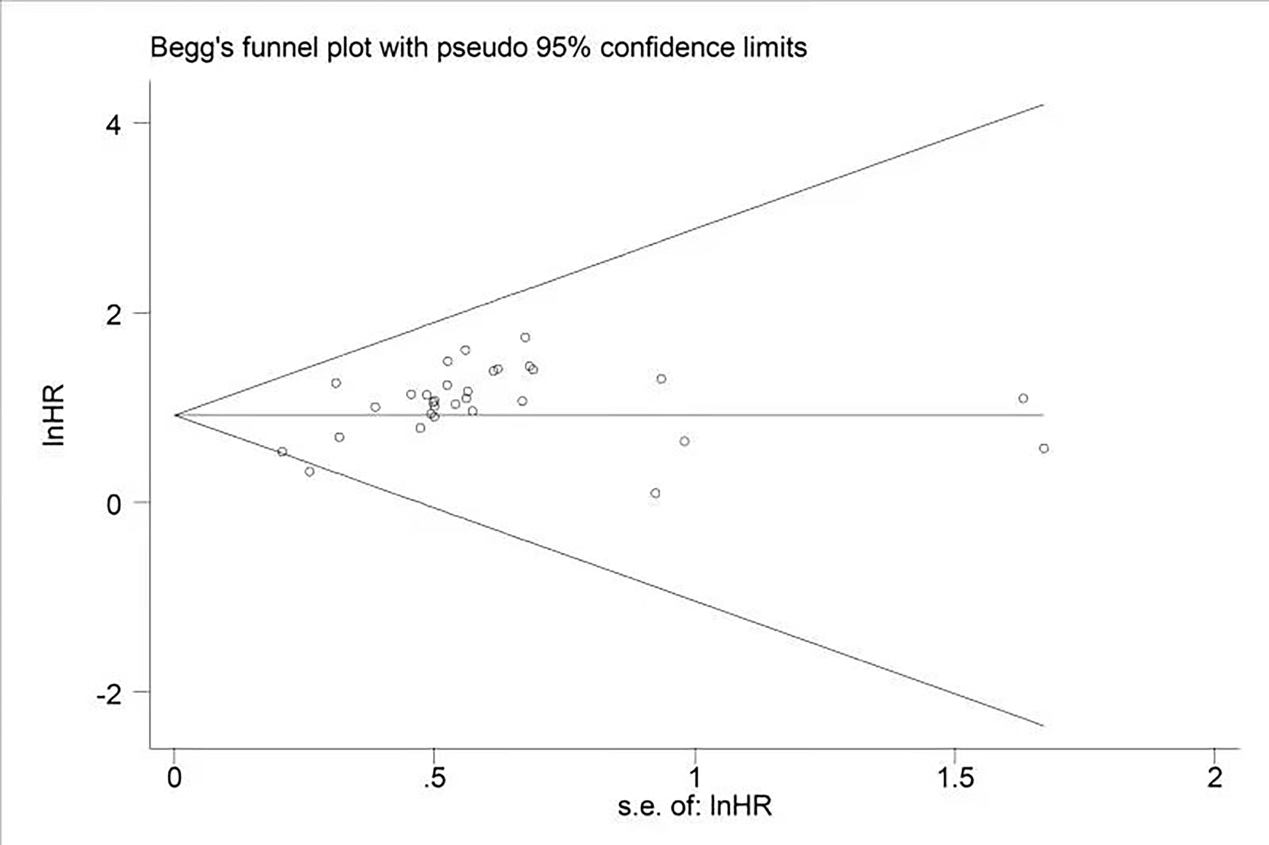


*Fig. S1 Funnel plots of the publication bias for recurrence-free survival (RFS)*

*
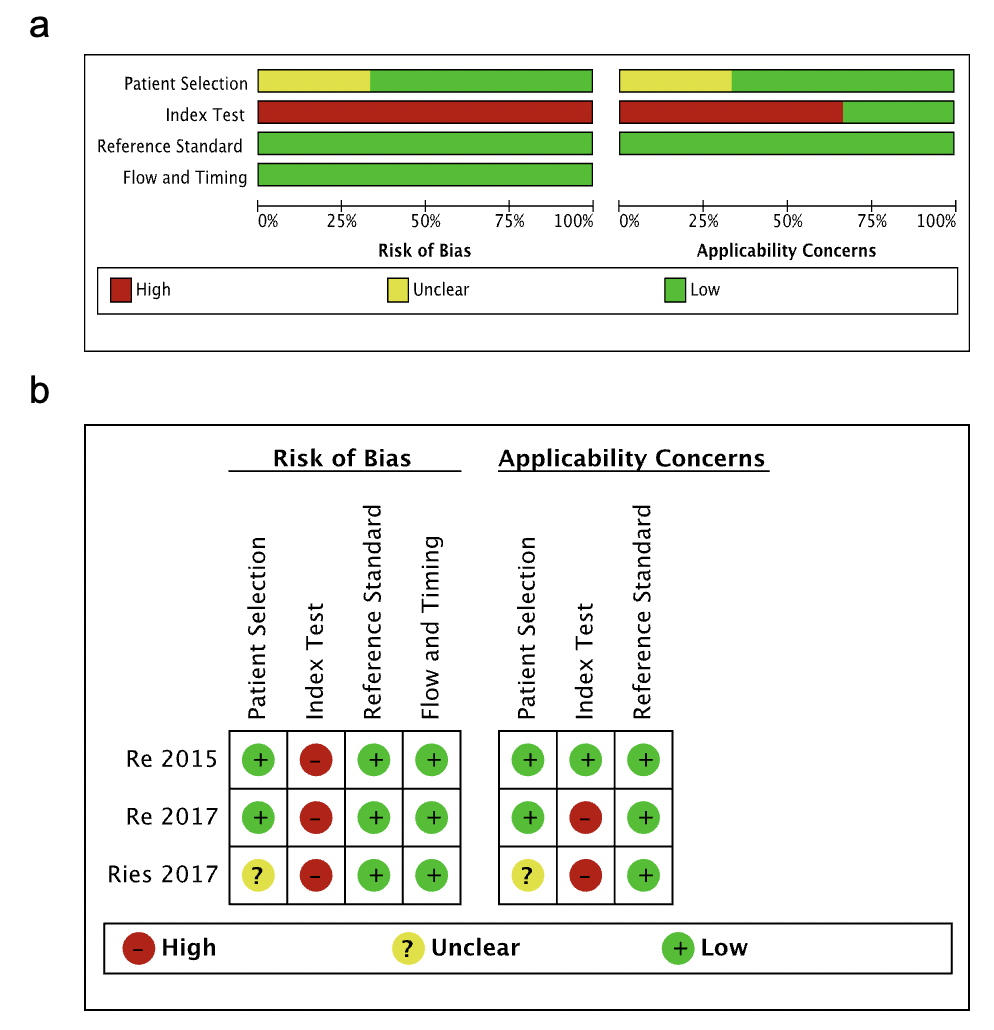
*

*Fig. S2 Methodological evaluation according to QUADAS-2 of the included studies (a) overall and (b) by study*


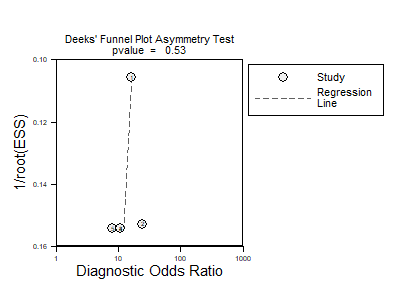


*Fig. S3 Deek’s Funnel plots of the publication bias for diagnostic accuracy of recurrence*

*
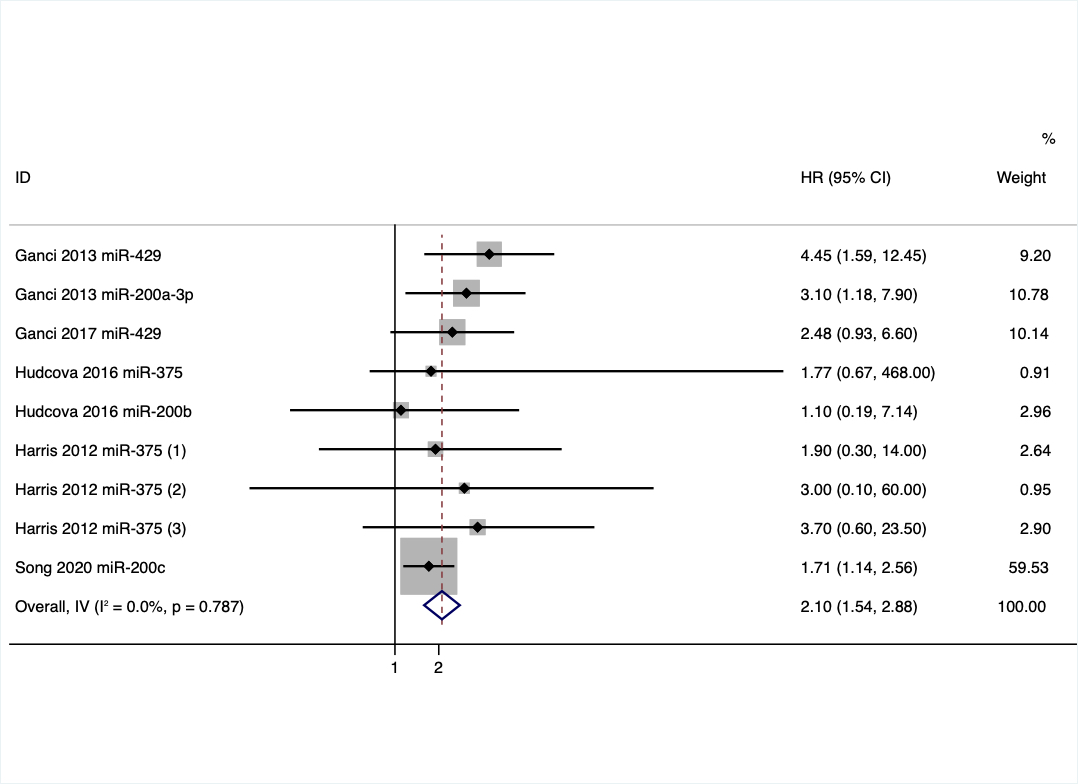
*

*Fig. S4* *Forest plot for the association between the expression level of microRNAs from microRNA-200 family and recurrence-free survival (RFS)*

Cao, L., Xie, B., Yang, X., Liang, H., Jiang, X., Zhang, D., et al. (2015). MiR-324-5p Suppresses Hepatocellular Carcinoma Cell Invasion by Counteracting ECM Degradation through Post-Transcriptionally Downregulating ETS1 and SP1. *PLoS One* 10(7)**,** e0133074. doi: 10.1371/journal.pone.0133074.

Cao, W., Zhao, Y., Wang, L., and Huang, X. (2019). Circ0001429 regulates progression of bladder cancer through binding miR-205-5p and promoting VEGFA expression. *Cancer Biomark* 25(1)**,** 101-113. doi: 10.3233/cbm-182380.

Cao, Z.H., Cheng, J.L., Zhang, Y., Bo, C.X., and Li, Y.L. (2017). MicroRNA‑375 inhibits oral squamous cell carcinoma cell migration and invasion by targeting platelet‑derived growth factor‑A. *Mol Med Rep* 15(2)**,** 922-928. doi: 10.3892/mmr.2016.6057.

Chang, R.M., Yang, H., Fang, F., Xu, J.F., and Yang, L.Y. (2014). MicroRNA-331-3p promotes proliferation and metastasis of hepatocellular carcinoma by targeting PH domain and leucine-rich repeat protein phosphatase. *Hepatology* 60(4)**,** 1251-1263. doi: 10.1002/hep.27221.

Chava, S., Reynolds, C.P., Pathania, A.S., Gorantla, S., Poluektova, L.Y., Coulter, D.W., et al. (2020). miR-15a-5p, miR-15b-5p, and miR-16-5p inhibit tumor progression by directly targeting MYCN in neuroblastoma. *Mol Oncol* 14(1)**,** 180-196. doi: 10.1002/1878-0261.12588.

Chen, H., Li, Z., Zhang, L., Zhang, L., Zhang, Y., Wang, Y., et al. (2020). MicroRNA-200c Inhibits the Metastasis of Triple-Negative Breast Cancer by Targeting ZEB2, an Epithelial-Mesenchymal Transition Regulator. *Ann Clin Lab Sci* 50(4)**,** 519-527.

Chen, J., Yan, D., Wu, W., Zhu, J., Ye, W., and Shu, Q. (2016). MicroRNA-130a promotes the metastasis and epithelial-mesenchymal transition of osteosarcoma by targeting PTEN. *Oncol Rep* 35(6)**,** 3285-3292. doi: 10.3892/or.2016.4719.

Chen, X., Luo, H., Li, X., Tian, X., Peng, B., Liu, S., et al. (2018a). miR-331-3p functions as an oncogene by targeting ST7L in pancreatic cancer. *Carcinogenesis* 39(8)**,** 1006-1015. doi: 10.1093/carcin/bgy074.

Chen, X., Zhao, M., Huang, J., Li, Y., Wang, S., Harrington, C.A., et al. (2018b). microRNA-130a suppresses breast cancer cell migration and invasion by targeting FOSL1 and upregulating ZO-1. *J Cell Biochem* 119(6)**,** 4945-4956. doi: 10.1002/jcb.26739.

Cheng, Y.X., Zhang, Q.F., Hong, L., Pan, F., Huang, J.L., Li, B.S., et al. (2016). MicroRNA-200b suppresses cell invasion and metastasis by inhibiting the epithelial-mesenchymal transition in cervical carcinoma. *Mol Med Rep* 13(4)**,** 3155-3160. doi: 10.3892/mmr.2016.4911.

Chung, T.K., Lau, T.S., Cheung, T.H., Yim, S.F., Lo, K.W., Siu, N.S., et al. (2012). Dysregulation of microRNA-204 mediates migration and invasion of endometrial cancer by regulating FOXC1. *Int J Cancer* 130(5)**,** 1036-1045. doi: 10.1002/ijc.26060.

Cui, F., Wang, S., Lao, I., Zhou, C., Kong, H., Bayaxi, N., et al. (2016). miR-375 inhibits the invasion and metastasis of colorectal cancer via targeting SP1 and regulating EMT-associated genes. *Oncol Rep* 36(1)**,** 487-493. doi: 10.3892/or.2016.4834.

De Cola, A., Lamolinara, A., Lanuti, P., Rossi, C., Iezzi, M., Marchisio, M., et al. (2018). MiR-205-5p inhibition by locked nucleic acids impairs metastatic potential of breast cancer cells. *Cell Death Dis* 9(8)**,** 821. doi: 10.1038/s41419-018-0854-9.

Doberstein, K., Bretz, N.P., Schirmer, U., Fiegl, H., Blaheta, R., Breunig, C., et al. (2014). miR-21-3p is a positive regulator of L1CAM in several human carcinomas. *Cancer Lett* 354(2)**,** 455-466. doi: 10.1016/j.canlet.2014.08.020.

Fan, X., Fang, X., Liu, G., Xiong, Q., Li, Z., and Zhou, W. (2019). MicroRNA-204 inhibits the proliferation and metastasis of breast cancer cells by targeting PI3K/AKT pathway. *J buon* 24(3)**,** 1054-1059.

Feng, H., Zhang, Z., Qing, X., French, S.W., and Liu, D. (2019). miR-186-5p promotes cell growth, migration and invasion of lung adenocarcinoma by targeting PTEN. *Exp Mol Pathol* 108**,** 105-113. doi: 10.1016/j.yexmp.2019.04.007.

Feng, S., Zhu, X., Fan, B., Xie, D., Li, T., and Zhang, X. (2016). miR‑19a‑3p targets PMEPA1 and induces prostate cancer cell proliferation, migration and invasion. *Mol Med Rep* 13(5)**,** 4030-4038. doi: 10.3892/mmr.2016.5033.

Feng, Y.G., Zhao, J.F., Xiao, L., Rao, W.Y., Ran, C., and Xiao, Y.H. (2018). MicroRNA-19a-3p suppresses invasion and metastasis of prostate cancer via inhibiting SOX4. *Eur Rev Med Pharmacol Sci* 22(19)**,** 6245-6251. doi: 10.26355/eurrev_201810_16031.

Gregory, P.A., Bert, A.G., Paterson, E.L., Barry, S.C., Tsykin, A., Farshid, G., et al. (2008). The miR-200 family and miR-205 regulate epithelial to mesenchymal transition by targeting ZEB1 and SIP1. *Nat Cell Biol* 10(5)**,** 593-601. doi: 10.1038/ncb1722.

Gu, C., Zhang, M., Sun, W., and Dong, C. (2019). Upregulation of miR-324-5p Inhibits Proliferation and Invasion of Colorectal Cancer Cells by Targeting ELAVL1. *Oncol Res* 27(5)**,** 515-524. doi: 10.3727/096504018x15166183598572.

Gu, J., Wang, G., Liu, H., and Xiong, C. (2018). SATB2 targeted by methylated miR-34c-5p suppresses proliferation and metastasis attenuating the epithelial-mesenchymal transition in colorectal cancer. *Cell Prolif* 51(4)**,** e12455. doi: 10.1111/cpr.12455.

Gulei, D., Magdo, L., Jurj, A., Raduly, L., Cojocneanu-Petric, R., Moldovan, A., et al. (2018). The silent healer: miR-205-5p up-regulation inhibits epithelial to mesenchymal transition in colon cancer cells by indirectly up-regulating E-cadherin expression. *Cell Death Dis* 9(2)**,** 66. doi: 10.1038/s41419-017-0102-8.

Guo, C., Zhao, D., Zhang, Q., Liu, S., and Sun, M.Z. (2018). miR-429 suppresses tumor migration and invasion by targeting CRKL in hepatocellular carcinoma via inhibiting Raf/MEK/ERK pathway and epithelial-mesenchymal transition. *Sci Rep* 8(1)**,** 2375. doi: 10.1038/s41598-018-20258-8.

Hou, X., Yang, L., Jiang, X., Liu, Z., Li, X., Xie, S., et al. (2019). Role of microRNA-141-3p in the progression and metastasis of hepatocellular carcinoma cell. *Int J Biol Macromol* 128**,** 331-339. doi: 10.1016/j.ijbiomac.2019.01.144.

Hu, W.B., Wang, L., Huang, X.R., and Li, F. (2019). MicroRNA-204 targets SOX4 to inhibit metastasis of lung adenocarcinoma. *Eur Rev Med Pharmacol Sci* 23(4)**,** 1553-1562. doi: 10.26355/eurrev_201902_17114.

Huang, D., Wang, F., Wu, W., Lian, C., and Liu, E. (2019). MicroRNA-429 inhibits cancer cell proliferation and migration by targeting the AKT1 in melanoma. *Cancer Biomark* 26(1)**,** 63-68. doi: 10.3233/cbm-190289.

Humphries, B., Wang, Z., Li, Y., Jhan, J.R., Jiang, Y., and Yang, C. (2017). ARHGAP18 Downregulation by miR-200b Suppresses Metastasis of Triple-Negative Breast Cancer by Enhancing Activation of RhoA. *Cancer Res* 77(15)**,** 4051-4064. doi: 10.1158/0008-5472.Can-16-3141.

Hur, K., Toiyama, Y., Takahashi, M., Balaguer, F., Nagasaka, T., Koike, J., et al. (2013). MicroRNA-200c modulates epithelial-to-mesenchymal transition (EMT) in human colorectal cancer metastasis. *Gut* 62(9)**,** 1315-1326. doi: 10.1136/gutjnl-2011-301846.

Jiang, H., Huang, G., Zhao, N., Zhang, T., Jiang, M., He, Y., et al. (2018a). Long non-coding RNA TPT1-AS1 promotes cell growth and metastasis in cervical cancer via acting AS a sponge for miR-324-5p. *J Exp Clin Cancer Res* 37(1)**,** 169. doi: 10.1186/s13046-018-0846-8.

Jiang, X.M., Yu, X.N., Liu, T.T., Zhu, H.R., Shi, X., Bilegsaikhan, E., et al. (2018b). microRNA-19a-3p promotes tumor metastasis and chemoresistance through the PTEN/Akt pathway in hepatocellular carcinoma. *Biomed Pharmacother* 105**,** 1147-1154. doi: 10.1016/j.biopha.2018.06.097.

Jimenez, L., Sharma, V.P., Condeelis, J., Harris, T., Ow, T.J., Prystowsky, M.B., et al. (2015). MicroRNA-375 Suppresses Extracellular Matrix Degradation and Invadopodial Activity in Head and Neck Squamous Cell Carcinoma. *Arch Pathol Lab Med* 139(11)**,** 1349-1361. doi: 10.5858/arpa.2014-0471-OA.

Kong, K.L., Kwong, D.L., Chan, T.H., Law, S.Y., Chen, L., Li, Y., et al. (2012). MicroRNA-375 inhibits tumour growth and metastasis in oesophageal squamous cell carcinoma through repressing insulin-like growth factor 1 receptor. *Gut* 61(1)**,** 33-42. doi: 10.1136/gutjnl-2011-300178.

Lang, Y., Xu, S., Ma, J., Wu, J., Jin, S., Cao, S., et al. (2014). MicroRNA-429 induces tumorigenesis of human non-small cell lung cancer cells and targets multiple tumor suppressor genes. *Biochem Biophys Res Commun* 450(1)**,** 154-159. doi: 10.1016/j.bbrc.2014.05.084.

Li, C., Ding, D., Gao, Y., and Li, Y. (2020). MicroRNA‑3651 promotes colorectal cancer cell proliferation through directly repressing T‑box transcription factor 1. *Int J Mol Med* 45(3)**,** 956-966. doi: 10.3892/ijmm.2020.4458.

Li, J., Xia, L., Zhou, Z., Zuo, Z., Xu, C., Song, H., et al. (2018a). MiR-186-5p upregulation inhibits proliferation, metastasis and epithelial-to-mesenchymal transition of colorectal cancer cell by targeting ZEB1. *Arch Biochem Biophys* 640**,** 53-60. doi: 10.1016/j.abb.2018.01.002.

Li, J., Yuan, J., Yuan, X., Zhao, J., Zhang, Z., Weng, L., et al. (2016). MicroRNA-200b inhibits the growth and metastasis of glioma cells via targeting ZEB2. *Int J Oncol* 48(2)**,** 541-550. doi: 10.3892/ijo.2015.3267.

Li, Q., Li, B., Li, Q., Wei, S., He, Z., Huang, X., et al. (2018b). Exosomal miR-21-5p derived from gastric cancer promotes peritoneal metastasis via mesothelial-to-mesenchymal transition. *Cell Death Dis* 9(9)**,** 854. doi: 10.1038/s41419-018-0928-8.

Li, X., Zhu, J., Liu, Y., Duan, C., Chang, R., and Zhang, C. (2019a). MicroRNA-331-3p inhibits epithelial-mesenchymal transition by targeting ErbB2 and VAV2 through the Rac1/PAK1/β-catenin axis in non-small-cell lung cancer. *Cancer Sci* 110(6)**,** 1883-1896. doi: 10.1111/cas.14014.

Li, Y., Guan, B., Liu, J., Zhang, Z., He, S., Zhan, Y., et al. (2019b). MicroRNA-200b is downregulated and suppresses metastasis by targeting LAMA4 in renal cell carcinoma. *EBioMedicine* 44**,** 439-451. doi: 10.1016/j.ebiom.2019.05.041.

Liao, G., Liu, X., Wu, D., Duan, F., Xie, X., Wen, S., et al. (2019). MORC2 promotes cell growth and metastasis in human cholangiocarcinoma and is negatively regulated by miR-186-5p. *Aging (Albany NY)* 11(11)**,** 3639-3649. doi: 10.18632/aging.102003.

Lin, H., Zhou, A.J., Zhang, J.Y., Liu, S.F., and Gu, J.X. (2018). MiR-324-5p reduces viability and induces apoptosis in gastric cancer cells through modulating TSPAN8. *J Pharm Pharmacol* 70(11)**,** 1513-1520. doi: 10.1111/jphp.12995.

Liu, H., Cheng, Y., Xu, Y., Xu, H., Lin, Z., Fan, J., et al. (2019a). The inhibition of tumor protein p53 by microRNA-151a-3p induced cell proliferation, migration and invasion in nasopharyngeal carcinoma. *Biosci Rep* 39(10). doi: 10.1042/bsr20191357.

Liu, M., Xiusheng, H., Xiao, X., and Wang, Y. (2016). Overexpression of miR-422a inhibits cell proliferation and invasion, and enhances chemosensitivity in osteosarcoma cells. *Oncol Rep* 36(6)**,** 3371-3378. doi: 10.3892/or.2016.5182.

Liu, N., Tang, L.L., Sun, Y., Cui, R.X., Wang, H.Y., Huang, B.J., et al. (2013). MiR-29c suppresses invasion and metastasis by targeting TIAM1 in nasopharyngeal carcinoma. *Cancer Lett* 329(2)**,** 181-188. doi: 10.1016/j.canlet.2012.10.032.

Liu, P.L., Liu, W.L., Chang, J.M., Chen, Y.H., Liu, Y.P., Kuo, H.F., et al. (2017). MicroRNA-200c inhibits epithelial-mesenchymal transition, invasion, and migration of lung cancer by targeting HMGB1. *PLoS One* 12(7)**,** e0180844. doi: 10.1371/journal.pone.0180844.

Liu, Z.M., Wu, Z.Y., Li, W.H., Wang, L.Q., Wan, J.N., and Zhong, Y. (2019b). MiR-96-5p promotes the proliferation, invasion and metastasis of papillary thyroid carcinoma through down-regulating CCDC67. *Eur Rev Med Pharmacol Sci* 23(8)**,** 3421-3430. doi: 10.26355/eurrev_201904_17706.

Lu, D., Tang, L., Zhuang, Y., and Zhao, P. (2018). miR-17-3P regulates the proliferation and survival of colon cancer cells by targeting Par4. *Mol Med Rep* 17(1)**,** 618-623. doi: 10.3892/mmr.2017.7863.

Lu, G., and Zhang, Y. (2020). Long non-coding RNA ATB promotes human non-small cell lung cancer proliferation and metastasis by suppressing miR-141-3p. *PLoS One* 15(2)**,** e0229118. doi: 10.1371/journal.pone.0229118.

Mei, J., Wang, D.H., Wang, L.L., Chen, Q., Pan, L.L., and Xia, L. (2018). MicroRNA-200c suppressed cervical cancer cell metastasis and growth via targeting MAP4K4. *Eur Rev Med Pharmacol Sci* 22(3)**,** 623-631. doi: 10.26355/eurrev_201802_14286.

Nagai, H., Hasegawa, S., Uchida, F., Terabe, T., Ishibashi Kanno, N., Kato, K., et al. (2018). MicroRNA-205-5p suppresses the invasiveness of oral squamous cell carcinoma by inhibiting TIMP‑2 expression. *Int J Oncol* 52(3)**,** 841-850. doi: 10.3892/ijo.2018.4260.

Qiu, M., Liang, Z., Chen, L., Tan, G., Wang, K., Liu, L., et al. (2015). MicroRNA-429 suppresses cell proliferation, epithelial-mesenchymal transition, and metastasis by direct targeting of BMI1 and E2F3 in renal cell carcinoma. *Urol Oncol* 33(7)**,** 332.e339-318. doi: 10.1016/j.urolonc.2015.03.016.

Samantarrai, D., and Mallick, B. (2017). miR-429 inhibits metastasis by targeting KIAA0101 in Soft Tissue Sarcoma. *Exp Cell Res* 357(1)**,** 33-39. doi: 10.1016/j.yexcr.2017.04.017.

Sarkar, A., Rahaman, A., Biswas, I., Mukherjee, G., Chatterjee, S., Bhattacharjee, S., et al. (2020). TGFβ mediated LINC00273 upregulation sponges mir200a-3p and promotes invasion and metastasis by activating ZEB1. *J Cell Physiol* 235(10)**,** 7159-7172. doi: 10.1002/jcp.29614.

Shi, J., Wang, H., Feng, W., Huang, S., An, J., Qiu, Y., et al. (2020). MicroRNA-130a targeting hypoxia-inducible factor 1 alpha suppresses cell metastasis and Warburg effect of NSCLC cells under hypoxia. *Life Sci* 255**,** 117826. doi: 10.1016/j.lfs.2020.117826.

Shi, L., Zhang, B., Sun, X., Lu, S., Liu, Z., Liu, Y., et al. (2014). MiR-204 inhibits human NSCLC metastasis through suppression of NUAK1. *Br J Cancer* 111(12)**,** 2316-2327. doi: 10.1038/bjc.2014.580.

Sun, J., Chen, Z., Xiong, J., Wang, Q., Tang, F., Zhang, X., et al. (2020). MicroRNA‑422a functions as a tumor suppressor in glioma by regulating the Wnt/β‑catenin signaling pathway via RPN2. *Oncol Rep* 44(5)**,** 2108-2120. doi: 10.3892/or.2020.7741.

Sun, Y., Shen, S., Liu, X., Tang, H., Wang, Z., Yu, Z., et al. (2014). MiR-429 inhibits cells growth and invasion and regulates EMT-related marker genes by targeting Onecut2 in colorectal carcinoma. *Mol Cell Biochem* 390(1-2)**,** 19-30. doi: 10.1007/s11010-013-1950-x.

Tan, T., Xu, X.H., Lu, X.H., and Wang, X.W. (2020). MiRNA-200a-3p suppresses the proliferation, migration and invasion of non-small cell lung cancer through targeting IRS2. *Eur Rev Med Pharmacol Sci* 24(2)**,** 712-720. doi: 10.26355/eurrev_202001_20050.

Tseng, H.H., Tseng, Y.K., You, J.J., Kang, B.H., Wang, T.H., Yang, C.M., et al. (2017). Next-generation Sequencing for microRNA Profiling: MicroRNA-21-3p Promotes Oral Cancer Metastasis. *Anticancer Res* 37(3)**,** 1059-1066. doi: 10.21873/anticanres.11417.

Wa, Q., Li, L., Lin, H., Peng, X., Ren, D., Huang, Y., et al. (2018). Downregulation of miR‑19a‑3p promotes invasion, migration and bone metastasis via activating TGF‑β signaling in prostate cancer. *Oncol Rep* 39(1)**,** 81-90. doi: 10.3892/or.2017.6096.

Wang, F., Li, Y., Zhou, J., Xu, J., Peng, C., Ye, F., et al. (2011). miR-375 is down-regulated in squamous cervical cancer and inhibits cell migration and invasion via targeting transcription factor SP1. *Am J Pathol* 179(5)**,** 2580-2588. doi: 10.1016/j.ajpath.2011.07.037.

Wang, H., Tang, C., Na, M., Ma, W., Jiang, Z., Gu, Y., et al. (2017). miR-422a Inhibits Glioma Proliferation and Invasion by Targeting IGF1 and IGF1R. *Oncol Res* 25(2)**,** 187-194. doi: 10.3727/096504016x14732772150389.

Wang, H., Zhu, Y., Zhao, M., Wu, C., Zhang, P., Tang, L., et al. (2013). miRNA-29c suppresses lung cancer cell adhesion to extracellular matrix and metastasis by targeting integrin β1 and matrix metalloproteinase2 (MMP2). *PLoS One* 8(8)**,** e70192. doi: 10.1371/journal.pone.0070192.

Wang, L., Yu, T., Li, W., Li, M., Zuo, Q., Zou, Q., et al. (2019a). The miR-29c-KIAA1199 axis regulates gastric cancer migration by binding with WBP11 and PTP4A3. *Oncogene* 38(17)**,** 3134-3150. doi: 10.1038/s41388-018-0642-0.

Wang, S., Han, H., Hu, Y., Yang, W., Lv, Y., Wang, L., et al. (2018). MicroRNA-130a-3p suppresses cell migration and invasion by inhibition of TBL1XR1-mediated EMT in human gastric carcinoma. *Mol Carcinog* 57(3)**,** 383-392. doi: 10.1002/mc.22762.

Wang, Y.Y., Yan, L., Yang, S., Xu, H.N., Chen, T.T., Dong, Z.Y., et al. (2019b). Long noncoding RNA AC073284.4 suppresses epithelial-mesenchymal transition by sponging miR-18b-5p in paclitaxel-resistant breast cancer cells. *J Cell Physiol* 234(12)**,** 23202-23215. doi: 10.1002/jcp.28887.

Wei, L.J., Bai, D.M., Wang, Z.Y., and Liu, B.C. (2019a). MicroRNA-375 accelerates the invasion and migration of colorectal cancer through targeting RECK. *Eur Rev Med Pharmacol Sci* 23(11)**,** 4738-4745. doi: 10.26355/eurrev_201906_18055.

Wei, S., Zheng, Y., Jiang, Y., Li, X., Geng, J., Shen, Y., et al. (2019b). The circRNA circPTPRA suppresses epithelial-mesenchymal transitioning and metastasis of NSCLC cells by sponging miR-96-5p. *EBioMedicine* 44**,** 182-193. doi: 10.1016/j.ebiom.2019.05.032.

Williams, L.V., Veliceasa, D., Vinokour, E., and Volpert, O.V. (2013). miR-200b inhibits prostate cancer EMT, growth and metastasis. *PLoS One* 8(12)**,** e83991. doi: 10.1371/journal.pone.0083991.

Wu, G., Zheng, H., Xu, J., Guo, Y., Zheng, G., Ma, C., et al. (2019). miR-429 suppresses cell growth and induces apoptosis of human thyroid cancer cell by targeting ZEB1. *Artif Cells Nanomed Biotechnol* 47(1)**,** 548-554. doi: 10.1080/21691401.2018.1564320.

Xiang, W., Lv, L., Zhou, G., Wu, W., Yuan, J., Zhang, C., et al. (2020). The lncRNA SNHG5-mediated miR-205-5p downregulation contributes to the progression of clear cell renal cell carcinoma by targeting ZEB1. *Cancer Med* 9(12)**,** 4251-4264. doi: 10.1002/cam4.3052.

Xie, N.N., Liu, Z.X., Wu, C., Wang, P.L., Song, G.T., and Chen, Z. (2018). MicroRNA-200c suppresses tumor metastasis in oral squamous carcinoma by inhibiting epithelial-mesenchymal transition. *Eur Rev Med Pharmacol Sci* 22(11)**,** 3415-3422. doi: 10.26355/eurrev_201806_15164.

Xue, H., and Tian, G.Y. (2018). MiR-429 regulates the metastasis and EMT of HCC cells through targeting RAB23. *Arch Biochem Biophys* 637**,** 48-55. doi: 10.1016/j.abb.2017.11.011.

Yang, J., Zhang, Z., Chen, C., Liu, Y., Si, Q., Chuang, T.H., et al. (2014). MicroRNA-19a-3p inhibits breast cancer progression and metastasis by inducing macrophage polarization through downregulated expression of Fra-1 proto-oncogene. *Oncogene* 33(23)**,** 3014-3023. doi: 10.1038/onc.2013.258.

Yang, S., Chen, B., Zhang, B., Li, C., Qiu, Y., Yang, H., et al. (2020a). miR‑204‑5p promotes apoptosis and inhibits migration of gastric cancer cells by targeting HER‑2. *Mol Med Rep* 22(4)**,** 2645-2654. doi: 10.3892/mmr.2020.11367.

Yang, S., Yang, R., Lin, R., and Si, L. (2019). MicroRNA-375 inhibits the growth, drug sensitivity and metastasis of human ovarian cancer cells by targeting PAX2. *J buon* 24(6)**,** 2341-2346.

Yang, X., Du, W.W., Li, H., Liu, F., Khorshidi, A., Rutnam, Z.J., et al. (2013). Both mature miR-17-5p and passenger strand miR-17-3p target TIMP3 and induce prostate tumor growth and invasion. *Nucleic Acids Res* 41(21)**,** 9688-9704. doi: 10.1093/nar/gkt680.

Yang, Y., Xia, S., Zhang, L., Wang, W., Chen, L., and Zhan, W. (2020b). MiR-324-5p/PTPRD/CEBPD axis promotes papillary thyroid carcinoma progression via microenvironment alteration. *Cancer Biol Ther* 21(6)**,** 522-532. doi: 10.1080/15384047.2020.1736465.

Yi, J., Jin, L., Chen, J., Feng, B., He, Z., Chen, L., et al. (2017a). MiR-375 suppresses invasion and metastasis by direct targeting of SHOX2 in esophageal squamous cell carcinoma. *Acta Biochim Biophys Sin (Shanghai)* 49(2)**,** 159-169. doi: 10.1093/abbs/gmw131.

Yi, L., Liu, M., and Tang, Z. (2017b). MicroRNA‑130a inhibits growth and metastasis of osteosarcoma cells by directly targeting ZEB1. *Mol Med Rep* 16(3)**,** 3606-3612. doi: 10.3892/mmr.2017.6968.

Yu, Y., Wang, Y., Xiao, X., Cheng, W., Hu, L., Yao, W., et al. (2019). MiR-204 inhibits hepatocellular cancer drug resistance and metastasis through targeting NUAK1. *Biochem Cell Biol* 97(5)**,** 563-570. doi: 10.1139/bcb-2018-0354.

Zeng, F., Xue, M., Xiao, T., Li, Y., Xiao, S., Jiang, B., et al. (2016). MiR-200b promotes the cell proliferation and metastasis of cervical cancer by inhibiting FOXG1. *Biomed Pharmacother* 79**,** 294-301. doi: 10.1016/j.biopha.2016.02.033.

Zhang, B., Cui, H., Sun, Y., Wang, X., Jia, Q., Li, J., et al. (2021). Up-regulation of miR-204 inhibits proliferation, invasion and apoptosis of gallbladder cancer cells by targeting Notch2. *Aging (Albany NY)* 13(2)**,** 2941-2958. doi: 10.18632/aging.202444.

Zhang, H., Chen, R., and Shao, J. (2020a). MicroRNA-96-5p Facilitates the Viability, Migration, and Invasion and Suppresses the Apoptosis of Cervical Cancer Cells byNegatively Modulating SFRP4. *Technol Cancer Res Treat* 19**,** 1533033820934132. doi: 10.1177/1533033820934132.

Zhang, J., Yang, Y., Yang, T., Yuan, S., Wang, R., Pan, Z., et al. (2015). Double-negative feedback loop between microRNA-422a and forkhead box (FOX)G1/Q1/E1 regulates hepatocellular carcinoma tumor growth and metastasis. *Hepatology* 61(2)**,** 561-573. doi: 10.1002/hep.27491.

Zhang, J.X., Mai, S.J., Huang, X.X., Wang, F.W., Liao, Y.J., Lin, M.C., et al. (2014). MiR-29c mediates epithelial-to-mesenchymal transition in human colorectal carcinoma metastasis via PTP4A and GNA13 regulation of β-catenin signaling. *Ann Oncol* 25(11)**,** 2196-2204. doi: 10.1093/annonc/mdu439.

Zhang, P., Lu, X., Shi, Z., Li, X., Zhang, Y., Zhao, S., et al. (2019). miR-205-5p regulates epithelial-mesenchymal transition by targeting PTEN via PI3K/AKT signaling pathway in cisplatin-resistant nasopharyngeal carcinoma cells. *Gene* 710**,** 103-113. doi: 10.1016/j.gene.2019.05.058.

Zhang, R., and Xia, T. (2017). Long non-coding RNA XIST regulates PDCD4 expression by interacting with miR-21-5p and inhibits osteosarcoma cell growth and metastasis. *Int J Oncol* 51(5)**,** 1460-1470. doi: 10.3892/ijo.2017.4127.

Zhang, X., Yu, X., Zhao, Z., Yuan, Z., Ma, P., Ye, Z., et al. (2020b). MicroRNA-429 inhibits bone metastasis in breast cancer by regulating CrkL and MMP-9. *Bone* 130**,** 115139. doi: 10.1016/j.bone.2019.115139.

Zhang, X., Zhang, L., Chen, M., and Liu, D. (2020c). miR-324-5p inhibits gallbladder carcinoma cell metastatic behaviours by downregulation of transforming growth factor beta 2 expression. *Artif Cells Nanomed Biotechnol* 48(1)**,** 315-324. doi: 10.1080/21691401.2019.1703724.

Zhao, C., Li, Y., Chen, G., Wang, F., Shen, Z., and Zhou, R. (2017). Overexpression of miR-15b-5p promotes gastric cancer metastasis by regulating PAQR3. *Oncol Rep* 38(1)**,** 352-358. doi: 10.3892/or.2017.5673.

Zhao, M., Zhang, M., Tao, Z., Cao, J., Wang, L., and Hu, X. (2020). miR-331-3p Suppresses Cell Proliferation in TNBC Cells by Downregulating NRP2. *Technol Cancer Res Treat* 19**,** 1533033820905824. doi: 10.1177/1533033820905824.

Zheng, S., Jiang, F., Ge, D., Tang, J., Chen, H., Yang, J., et al. (2019). LncRNA SNHG3/miRNA-151a-3p/RAB22A axis regulates invasion and migration of osteosarcoma. *Biomed Pharmacother* 112**,** 108695. doi: 10.1016/j.biopha.2019.108695.

Zhu, Y., Bo, H., Chen, Z., Li, J., He, D., Xiao, M., et al. (2020). LINC00968 can inhibit the progression of lung adenocarcinoma through the miR-21-5p/SMAD7 signal axis. *Aging (Albany NY)* 12(21)**,** 21904-21922. doi: 10.18632/aging.104011.
